# Desmoid fibromatosis of the cruris: A rare case report

**DOI:** 10.1016/j.radcr.2025.09.039

**Published:** 2025-10-11

**Authors:** Nuciana Siti Andrianti, Ahmad Fitrah, Aviyani, Bethy S. Hernawa

**Affiliations:** a Department of Radiology, Faculty of Medicine, Universitas Padjadjaran, Dr. Hasan Sadikin General Hospital, Bandung, Indonesia; b Department of Anatomical Pathology, Faculty of Medicine, Universitas Padjadjaran, Dr. Hasan Sadikin General Hospital, Bandung, Indonesia

**Keywords:** Desmoid Fibromatosis, Magnetic resonance imaging (MRI)

## Abstract

Desmoid Fibromatosis (DF) is a locally aggressive connective tissue neoplasm that arises within the musculoaponeurotic structures. It is also referred to by various terms, including *aggressive fibromatosis, deep fibromatosis, musculoaponeurotic fibromatosis*, and *desmoid tumor*. DF is a rare tumor, with a reported incidence of 2–4 cases per million population, accounting for approximately 0.03% of all neoplasms. It most commonly occurs between the ages of 15 and 60 years and tends to be more prevalent in females. Although DF can develop in various anatomical locations, it is most frequently observed in the extremities, abdominal wall, and intra-abdominal mesentery. Despite lacking metastatic potential, DF has a high propensity for local recurrence. Due to these biological characteristics, DF is currently classified as an “intermediate, locally aggressive” tumor according to the World Health Organization (WHO) classification of soft tissue tumors. This report presents a clinical case of desmoid fibromatosis.

## Introduction

Desmoid fibromatosis (DF), also known as aggressive fibromatosis or desmoid tumor, is a rare, benign soft tissue neoplasm arising from musculoaponeurotic structures [[1], [2]]. Despite its benign histological appearance and lack of metastatic potential, DF demonstrates locally aggressive behavior and is associated with a high risk of local recurrence [[1], [3]]. The annual incidence is estimated at 2-4 cases per million population, accounting for approximately 0.03% of all neoplasms [1]. DF most commonly affects individuals between 15 and 60 years of age and shows a higher prevalence in females [3]. However, pediatric cases, though uncommon, have been reported [[1], [4]].

The etiology of DF remains uncertain. Most cases are sporadic and are frequently associated with mutations in the *CTNNB1* gene encoding β-catenin [2]. DF is also known to occur in patients with familial adenomatous polyposis (FAP), particularly in association with Gardner syndrome [1]. Depending on the anatomical location, DF may be classified as abdominal, intra-abdominal, or extra-abdominal [3].

Magnetic Resonance Imaging (MRI) plays a pivotal role in the diagnosis, characterization, and treatment planning of DF due to its superior soft tissue resolution and ability to assess local infiltration [[3], [4]]. Histopathological examination, supported by immunohistochemistry, remains the gold standard for definitive diagnosis [1].

This case report presents a rare occurrence of DF in a three-year-old boy, highlighting the clinical presentation, radiologic features, and pathological findings that led to the final diagnosis.

## Case report

### Case presentation

The patient is a three-year-old boy who presented with a mass on his right leg that had been present for nine months prior to hospital admission. The mass was described as appearing suddenly, initially the size of a chicken egg, and had not shown progressive enlargement. The patient did not report any pain and was able to walk normally.

On physical examination, a mass approximately 10 cm in size was observed, with an overlying superficial skin lesion. The mass was non-tender to palpation. Neurological examination revealed no abnormalities. The patient was able to move his leg actively and passively without limitation. There was no history of previous trauma or fracture involving the tibia.

### Diagnostic evaluation

Subsequent investigations included anteroposterior and lateral plain radiographs of the right lower leg (cruris), magnetic resonance imaging (MRI), histopathological examination, and immunohistochemistry. All examinations were conducted at Hasan Sadikin General Hospital, Bandung.

**Fig 1:**
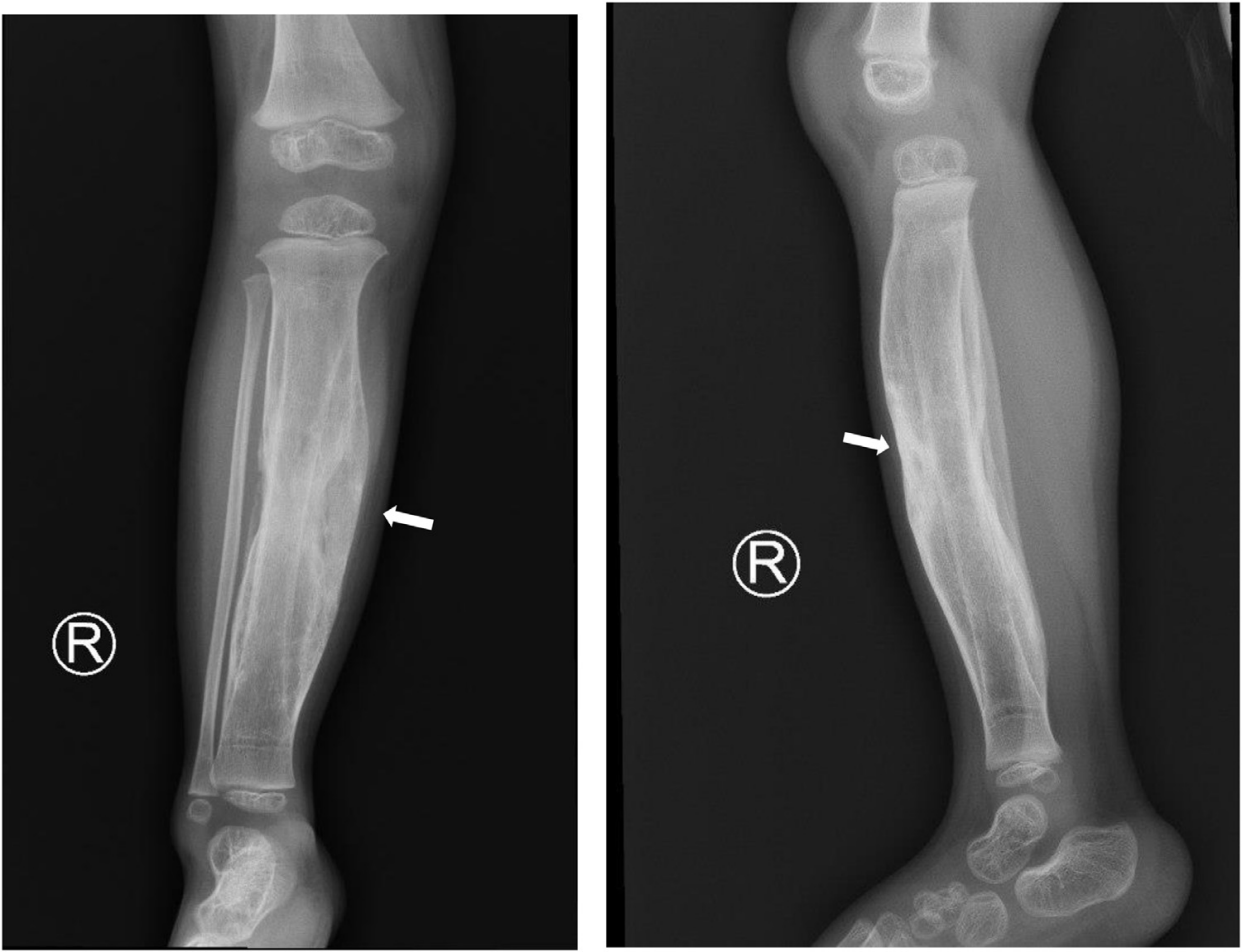
X-ray of the patient shows a soft tissue mass in the right cruris with peripheral calcification (arrow) causing compression and separation between the tibia and fibula.

Plain radiographs revealed a soft tissue density mass with peripheral calcifications, widening of the tibiofibular distance, and a solid periosteal reaction suggestive of periostitis (Fig. 1).

MRI of the right cruris was recommended. MRI examination revealed a relatively well-defined, irregular-margin, inhomogeneous mass measuring approximately 2.74 × 1.77 × 13.42 cm located in the anteromedial soft tissue of the right cruris, appearing to infiltrate the right tibial bone with associated bone marrow replacement.

**Fig. 2:**
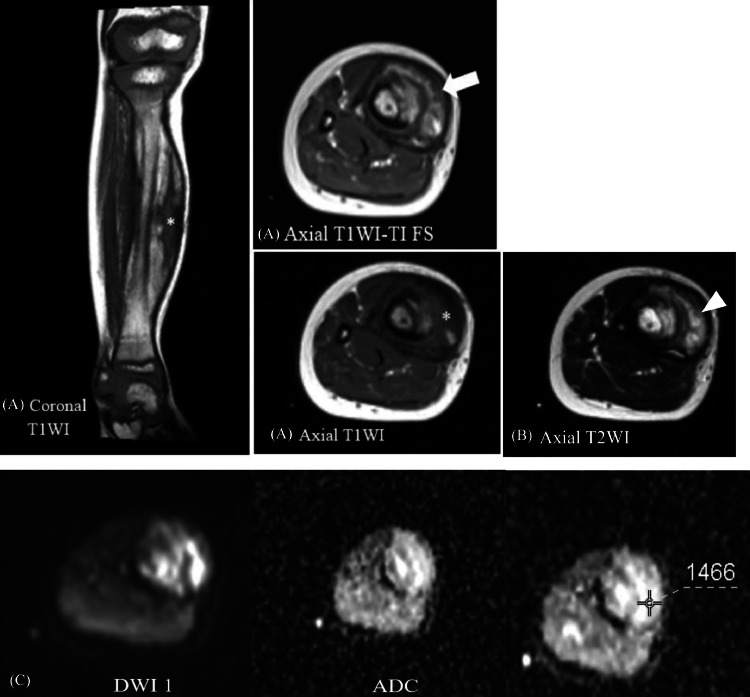
(A) axial and coronal T1WI MRI image show soft tissue mass with a relatively well-defined, irregular-margin, inhomogeneous soft tissue mass (*). The lesion shows inhomogeneous isointense to partially hyperintense signals on T1WI with fat saturation/T1 FS (arrow), (B) heterogeneous hyperintensity on axial T2WI (arrowhead), (C) no restricted diffusion on DWI-ADC (ADC: 1.493 × 10⁻³ mm²/s).

The lesion showed inhomogeneous isointensee signals with partially hyperintense areas on T1-weighted imaging (T1WI), inhomogeneous hyperintensity on T2-weighted imaging (T2WI), no restricted diffusion on DWI-ADC, an ADC value of 1.493 × 10⁻³ mm²/s and Post-contrast imaging demonstrated inhomogeneous enhancement (Fig. 2). The right fibula was displaced laterally, although its size and shape remained within normal limits. The visualized muscle and nerve structures appeared unremarkable. The MRI findings were supportive of a diagnosis of desmoid fibromatosis (DF).

**Fig. 3:**
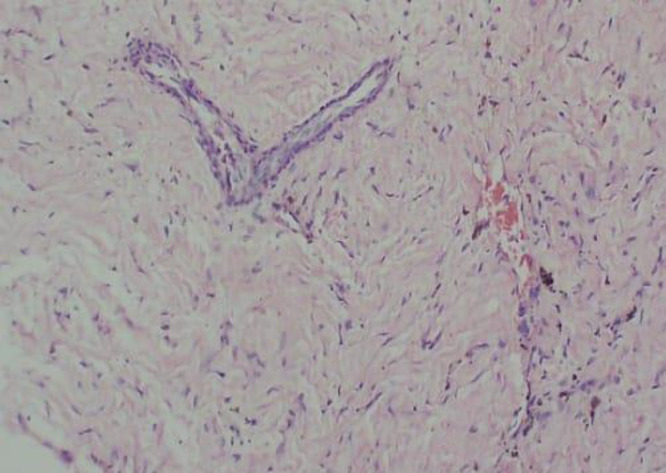
Histopathology picture shows a small tumor mass composed of round to oval and spindle-shaped cells was noted, demonstrating hyperplastic growth. Some cells showed a wavy pattern and were relatively monomorphic. Neovascularization was evident, with hyperplastic endothelial cells having nuclei within normal limits. Hemosiderin pigment and hemosiderin-laden macrophages were also present.

**Fig. 4:**
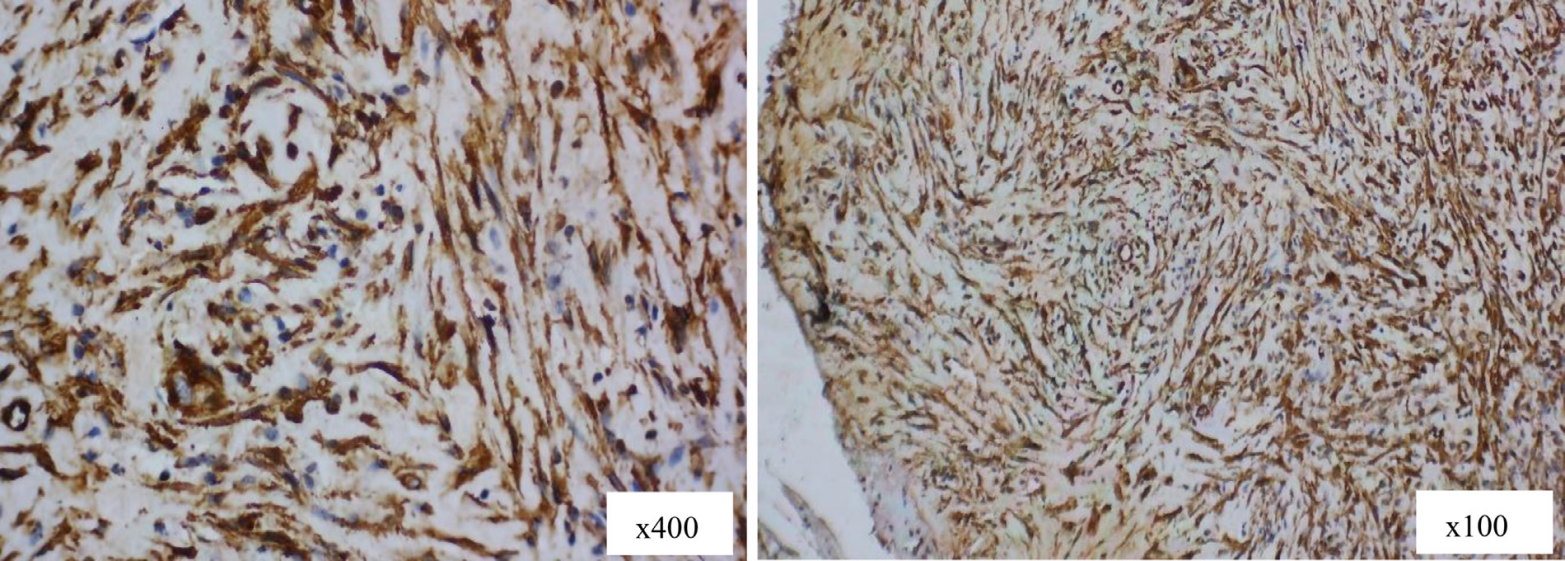
Immunohistochemical staining demonstrates positive smooth muscle actin (SMA) expression (IHC stain, ×400 and x100).

Histopathological examination provided a preliminary diagnosis of neurofibroma of the right tibial diaphysis, with a differential diagnosis of desmoid fibromatosis (Fig. 3). Immunohistochemical analysis showed positive staining for SMA (Fig. 4), and negative results for S100, CD34, Desmin, and CD117, supporting a final diagnosis of desmoid fibromatosis.

A diagnostic incisional biopsy was performed, and based on the histopathological and immunohistochemical findings, the diagnosis of desmoid fibromatosis was established. No surgical excision was undertaken, and the patient was managed conservatively with observation.

## Discussion

Desmoid fibromatosis (DF) is a mesenchymal neoplasm characterized by local invasiveness without metastatic potential [[1], [3]]. Despite its inability to metastasize, this tumor can result in significant morbidity and, in some cases, mortality. There is currently no standardized treatment approach for DF. The tumor is known for its high local recurrence rate following surgical resection [1]. The World Health Organization (WHO) classifies DF as an “intermediate, locally aggressive” soft tissue tumor [1]. DF may also present as multifocal lesions within the same limb or anatomical region [3].

The etiology of DF remains unclear. Most desmoid tumors occur sporadically, and approximately 85% of these tumors harbor mutations in the *CTNNB1* gene, which encodes β-catenin [2]. DF is also more frequently observed in association with familial adenomatous polyposis (FAP) [1]. The reported incidence of DF is 2-4 cases per million population, accounting for approximately 0.03% of all neoplasms [1]. It is more common in females than in males and predominantly affects individuals between 15 and 60 years of age [3]. Desmoid tumors can arise in abdominal, intra-abdominal, or extra-abdominal locations [3]. In this case, DF occurred in a three-year-old boy, which is relatively rare [[1], [4]].

Plain radiography plays a limited role in the diagnosis and management of DF. The most commonly utilized imaging modalities for DF are computed tomography (CT) and magnetic resonance imaging (MRI), while ultrasonography is useful in selected cases [[3], [4]]. Ultrasonography is particularly effective for visualizing tumors located in the abdominal wall and extremities. CT is commonly employed to detect intra-abdominal lesions [3]. The imaging characteristics of DF on various modalities, particularly MRI, strongly reflect its histological composition—namely, spindle cells, myxoid matrix, and surrounding collagenous stroma [3].

MRI is the preferred modality for evaluating DF due to its superior soft tissue resolution, especially for extra-abdominal lesions located in the extremities, head and neck, abdominal wall, and thoracic wall [3]. DF typically appears as a mass with low signal intensity on both T1- and T2-weighted images, due to its dense cellular composition. Gadolinium-enhanced T1-weighted imaging may show variable enhancement—ranging from homogeneous to heterogeneous, or even minimal [[3], [4]].

In the present case, MRI revealed an inhomogeneous mass. Recent studies have reported that the mean apparent diffusion coefficient (ADC) values in DF are significantly higher than those observed in malignant soft tissue sarcomas, indicating that diffusion-weighted imaging (DWI) may aid in differentiating DF from malignant tumors [4]. One study reported the average ADC value of fibromatosis masses to be approximately 1.31 ± 0.245 × 10^−^³ mm²/s. In this case, the measured ADC value was 1.493 × 10^−^³ mm²/s. This finding was subsequently confirmed through anatomical pathology, which remains the gold standard for diagnosing desmoid fibromatosis [1].

In this patient, there was no history of trauma or fracture, and the periosteal reaction with tibial deformity was most consistent with a secondary change due to chronic tumor infiltration [3]. The tumor originated from the anteromedial soft tissue of the cruris. Imaging demonstrated cortical involvement and bone marrow replacement of the tibia, indicating secondary invasion of the bone. Therefore, the tumor did not arise primarily from the tibia but extended into it, and was not entirely separate from the bone [3].

After the diagnostic incisional biopsy, no surgical excision was performed. Given the patient’s young age, preserved limb function, and the locally aggressive nature of desmoid fibromatosis, conservative observation was selected in accordance with current consensus guidelines [[5], [6], [7]]. During follow-up, the patient remained clinically stable without tumor progression, and mobility was preserved. This case illustrates that active surveillance may be an appropriate initial strategy in pediatric desmoid fibromatosis to minimize morbidity while maintaining quality of life [[6], [7]].

## Conclusion

Imaging plays a critical role in assessing the extent of desmoid fibromatosis, identifying involved or infiltrated structures, and guiding clinical decision-making. MRI is particularly effective in delineating tumor boundaries and evaluating its relationship with surrounding anatomical structures. In this case, imaging findings were consistent with histopathological examination, confirming the diagnosis of desmoid fibromatosis. Given the patient’s age, preserved limb function, and stable disease course, conservative observation was adopted as the management strategy. This case highlights both the diagnostic value of imaging and the importance of individualized treatment planning in desmoid fibromatosis, particularly in pediatric patients, where active surveillance can be prioritized to minimize morbidity while maintaining quality of life.

## Patient consent

This is to state that I give my full permission for the publication, reproduction, broadcast and other use of photographs, recordings and other audio-visual material of myself (including of my face) and textual material (case histories) in all editions of the above-named product and in any other publication (including books, journals, CD-ROMs, online and internet), as well as in any advertising or promotional material for such product or publications.

I declare, in consequence of granting this permission, that I have no claim on ground of breach of confidence or any other ground in any legal system against—and its agents, publishers, successors and assigns in respect of such use of the photograph(s) and textual material (case histories).

I hereby agree to release and discharge, and any editors or other contributors and their agents, publishers, successors and assigns from any and all claims, demands or causes of action that I may now have or may hereafter have for libel, defamation, invasion of privacy, copyright or moral rights or violation of any other rights arising out of or relating to any use of my image or case history.
